# Music intervention during non-stress test and its effects on maternal anxiety, maternal vital signs and fetal parameters: A quasi-experimental study

**DOI:** 10.18332/ejm/202215

**Published:** 2025-06-02

**Authors:** Manolis Astrinakis, Pinelopi Varela, Christina Nanou, Victoria Vivilaki, Anna Deltsidou

**Affiliations:** 1 Department of Midwifery, University of West Attica, Athens, Greece; 2 General Hospital of Athens ‘Alexandra’, Department of Midwifery, University of West Attica, Athens, Greece

**Keywords:** non-stress test, music intervention, anxiety, fetal heart rate, blood pressure, midwives

## Abstract

**INTRODUCTION:**

There is limited research on the impact of music intervention during the non-stress test. More investigation is required on whether and how music impacts non-stress test results and pregnant women’s mood. The current study aimed to examine the effect of the non-stress test on pregnant women’s anxiety levels and the effect of music on maternal anxiety levels, pregnant women’s vital signs, and fetal parameters.

**METHODS:**

A quasi-experimental study of two phases, involving one group, with a pre-and post-test, was performed at a private maternity and birth preparation center in Greece. Thirty-eight participants completed a scale for anxiety measurement (STAI) during their late third trimester; their vital signs were obtained, and the fetal parameters were recorded during the two phases of the study. During Phase 2, the music intervention, which referred to the exposure of pregnant women to musical stimuli during the non-stress test (NST), was carried out.

**RESULTS:**

Participants’ state anxiety with (mean=27.87, SD=4.55) or without music intervention (mean=31.16, SD=7.74) showed a significant score reduction after the completion of the NST (p=0.009), which was significantly greater with the music intervention (p<0.001). Levels of trait anxiety before the NST (mean=39.66, SD=5.44) and after its completion (mean=38.00, SD=5.39) showed a significant score reduction when there was music intervention (p<0.001). At the NST’s twelfth minute, participants’ systolic blood pressure was significantly lower when there was the music intervention (mean=93.2, SD=16.4 vs mean=99.5, SD=10.7, p=0.030), as well as at twenty minutes (mean=93, SD=8 vs mean=100, SD=9.7, p<0.001). Participants’ heart rate did not differ significantly between the examination with music (mean=85.9, SD=10.4) or without music (mean=84.9, SD=11.4) at baseline (p=0.506) or at other periods. Fetal movements increased significantly more in the last ten minutes of the NST compared to the first ten minutes, only when the NST was performed without music intervention (p=0.048). Accelerations were similar regardless of the presence or absence of music in the first ten minutes (p=0.235) and the last ten minutes (p=0.128), but they were increased significantly more in the last ten minutes of the NST compared to the first ten minutes, only when the NST was performed without music intervention (p=0.019).

**CONCLUSIONS:**

Maternal anxiety levels decreased following both the music intervention and the completion of the non-stress test. Music affected blood pressure and maintained stable fetal movements and accelerations. The preliminary findings of the present nonrandomized, quasi-experimental study, with one pre- and post-test group, indicate that music may be a potentially available option in midwifery.

## INTRODUCTION

Research is being conducted to investigate how music interventions, strategies, and practices impact people’s physical, mental, and emotional health. Musical vibrations can release hormones and activate the autonomic nervous system^1^. Previous research showed that music affected neurotransmitters, cytokines, hormones, immunoglobulins, and psychological responses in various ways^2^. Studies suggested that classical music with soft rhythms may decrease blood pressure and heart rate, deepen breathing, reduce stress, and lessen the need for medications^3,4^. Research has shown that incorporating music into healthcare can reduce pain, stress, and worries^5,6^. Additionally, systematic reviews have shown that listening to music has positive effects on anxiety in people with coronary heart disease or cancer^7,8^. The sympathetic nervous system may be suppressed by listening to music, resulting in lower cortisol levels and activating brain areas associated with emotional experiences and anxiety-modifying activity^2,9^.

Music interventions have also been investigated in studies related to the midwifery field. A recent systematic review and meta-analysis showed that music interventions during pregnancy may decrease antenatal maternal anxiety^10^. An earlier study also found that this anxiety reduction was observed in women with low- or high-risk pregnancies^11^. Listening to music during labor may help shorten the length of the first stage of labor as well as regulate pain perception, stress, and anxiety^12-14^. According to an RCT, participants who listened to music chosen independently over speakers, experienced much less pain and anxiety during labor compared to those who did not^15^. The use of music before, during, and after cesarean section appeared to improve the pregnant woman’s heart rate^16^ as well as decrease post-operative pain and maternal anxiety, or both^17-19^.

Overall, considerable evidence supports the positive effects of music-based interventions, approaches, and practices throughout the perinatal period. It is also noteworthy that researchers’ interest in the impact of music during the antenatal period is growing and has expanded to include the effects of music on non-invasive prenatal testing, such as the non-stress test (NST)^12,20,21^. Even though the NST is a painless procedure and the most widely used technique for assessing fetal well-being during the prenatal period, researchers are interested in learning whether its results are differential when combined with music or impact pregnant women’s mood^12,20,21^. Studies so far have investigated whether listening to music during the NST modifies pregnant women’s anxiety levels and their vital signs or has an effect on NST’s fetal parameters^12,20-22^. Due to the limited number of these studies and the lack of continuous consistency in their findings, the authors highlighted the need for more research on the topic^20-22^. Therefore, the present study aimed at examining the effect of the NST on pregnant women’s anxiety levels and the effect of music during the NST on maternal anxiety levels, pregnant women’s vital signs, and fetal parameters.

## METHODS

### Study design

**Figure 1:**
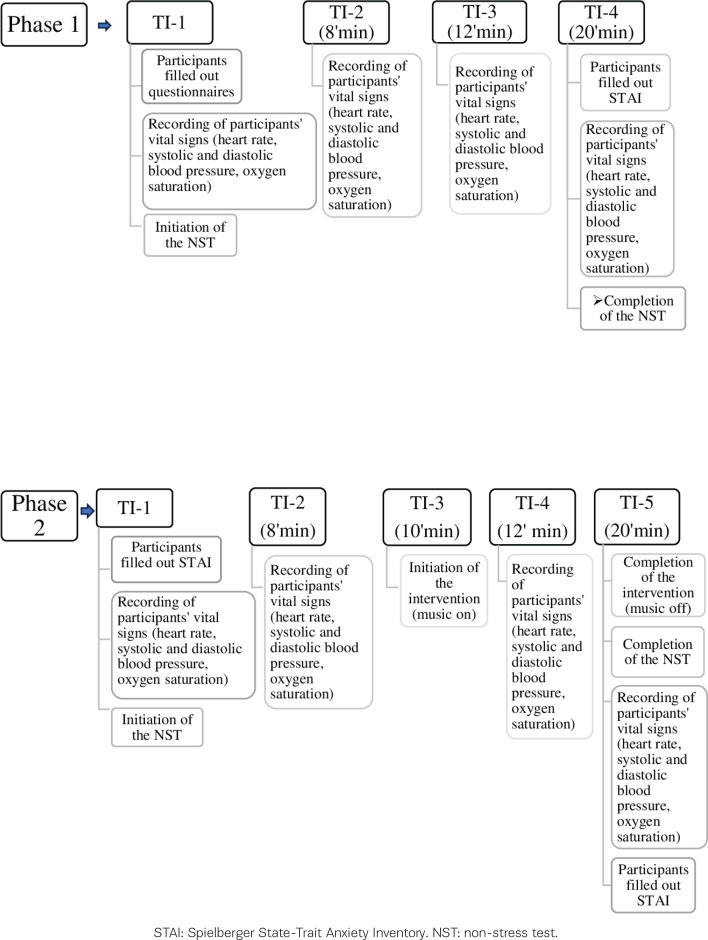
Procedures of the two phases of the study and the study's time intervals

The present study was quasi-experimental, with one group of pregnant women undergoing a pre-test and post-test. It consisted of two phases, during which the NSTs were performed. The study’s first phase (Phase 1) included four time periods. The first-time interval (Phase 1-TI-1) refers to the period just before the initiation of the NST. During this time, the participants filled in questionnaires, and after their vital signs were obtained, the NST was begun. The measurement of the pregnant women vital signs was repeated at the second and third-time intervals (Phase 1-TI-2 and Phase 1-TI-3), established at the eighth and twelfth minutes of the examination, respectively. The fourth and final time interval (Phase 1-TI-4) of Phase 1 was set at the twentieth minute with the completion of the NST, where the measurement of the vital signs was repeated, and the participants filled in the STAI scale again. Phase 2 of the study included five time periods when the intervention was carried out. Phase 2-TI-1 refers to the period just before the start of the NST. The participants completed the STAI scale, and after their vital signs were obtained, the NST was initiated. The second time interval (Phase 2-TI-2) was set at the eighth minute of the examination, and the vital signs were measured again. The tenth minute of the examination, when the intervention began, was established as the third time interval (Phase 2-TI-3). The fourth interval (Phase 2-TI-4) was set at the twelfth minute, where the intervention was continued, and the measurement of vital signs was repeated. The fifth and final time interval (Phase 2-TI-5) of Phase 2 was set at the twentieth minute with the completion of the NST when the interruption of the intervention took place, the measurement of the vital signs was repeated, and the participants filled in the STAI scale again (Figure 1). NST findings were divided into the first and second tenth minutes for comparison between the two phases since the intervention during Phase 2 started at the tenth minute of the recording. The Transparent Reporting of Evaluations with Non-randomized Designs (TREND) statement checklist was followed for the current study^23^.

### Setting

The study was conducted at one private maternity and birth preparation center in Chania, which collaborates with public and private maternity clinics. Chania, the second-largest city on the island of Crete, has approximately 110646 inhabitants and approximately 1627 births annually.

### Participants

Participants were approached during prenatal visits at the maternity and birth preparation center in the third trimester of pregnancy to determine their eligibility and interest in participating in the study. Eligible pregnant women were aged >18 years, fluent in Greek, had low-risk pregnancies, were in the late third trimester of their pregnancies, without any physical or mental disorder, and resided in the greater Chania region. Pregnant women who would not have enough time to complete the study’s stages, who needed immediate clinical attention, or who had an impending birth were excluded. All data were collected at the specific maternity and birth preparation center. Each pregnant woman received complete verbal and written information about all stages of the study. Participants’ involvement in the study was initiated after they provided completed and signed informed consent to the principal researcher.

**Figure 2:**
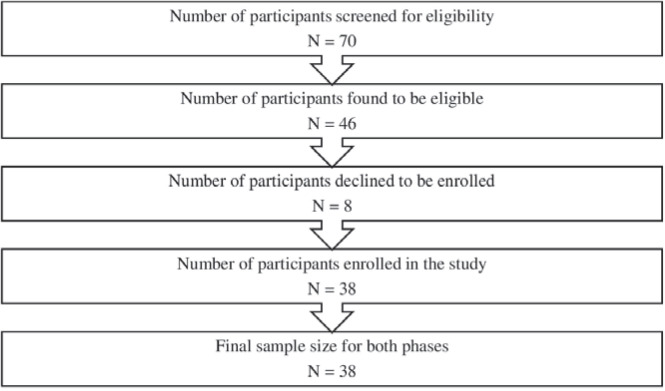
Participants flowchart

Convenience sampling was used to select eligible pregnant women. Seventy participants were screened for eligibility between January and April 2022, and 46 were found eligible. After removing those who declined to participate, the final sample size of the study consisted of 38 pregnant women, i.e. one group with a pre-test and post-test (Figure 2). The participation rate was 82%, which is acceptable according to the literature^24^. None of the participants dropped out of either research period; hence, no data were missing.

### Intervention

The intervention in this study refers to the exposure of pregnant women to musical stimuli during the NST. The setting of the intervention was one private maternity and birth preparation center. All participants received comprehensive information from the principal researcher (MA) on all study phases and all required interventional procedures. The most substantial incentive used was the information given to pregnant women about the sensation of relaxation they would feel by listening to music during the performance of NST. All phases of the study were overseen by the principal researcher (MA), who is trained in the administration of psychometric scales and who also implemented the intervention. The questionnaires that the pregnant women filled in, as well as the forms with the findings of the measurements obtained by the principal investigator (MA), were collected and archived by a midwife who was a staff member. Phase 2-TI-3, the tenth minute of the NST, served as the starting point for the intervention. The musical stimuli were the listening of the classical piece *Por ti volare*, instrumental violin, at a speed of 76 bpm, and sound volume at 65 dB from an audio player. The music’s volume (dB) was measured at the height of the center of the pregnant woman’s chest to avoid any volume loss due to the distance from the source and to provide an objective measurement. Two minutes after the beginning of the intervention (Phase 2-TI-4), the measurement of the pregnant woman’s vital signs was obtained. The duration of the intervention was ten minutes, and its finishing point coincides with the final stage of the study (Phase 2-TI-5). The participants were informed to refrain from consuming anything other than water for an hour before and during the NST.

### Outcomes and data collection

The mental outcome, i.e. the pregnant woman’s anxiety level, was assessed using the STAI scale. Clinical outcomes, i.e. the pregnant woman’s vital signs [heart rate (HR), systolic blood pressure (SBP), diastolic blood pressure (DBP), and oxygen saturation (SpO2)] and the fetal parameters, were recorded in a typical form for outcomes.

Pre-test data were collected during Phase 1 of the study when the participants were in the 37th to 41st weeks of gestation. Post-test data were collected ten days after the completion of Phase 1, i.e. during Phase 2. All data were collected between January and April 2022.

In Phase 1 (TI-1) of the study, all the participants completed a questionnaire developed for the present study that included questions on demographic characteristics, obstetric and medical history, pregnancy and breastfeeding intention, information regarding the type of upcoming birth, and attendance at prenatal classes.

The Spielberger State-Trait Anxiety Inventory (STAI) was administered to the participants for the assessment of their anxiety levels. Its completion occurred before and after the performance of the NST (Phase 1: TI-1 and TI-4; Phase 2: TI-1 and TI-5). Two subscales comprise the STAI. The state subscale assesses anxiety at the moment of the evaluation, which may change over time. The trait subscale measures anxiety as a persistently consistent personal trait. Twenty statements on each subscale are rated on a 4-point Likert scale from 1 to 4. For each subscale, the overall score ranges from 20 to 80, with higher scores showing higher anxiety levels^25^. The Greek version of the STAI was used, with a Cronbach’s alpha of 0.93 for the state subscale and 0.92 for the trait subscale^26^. Within-sample internal consistency was considered with a Cronbach’s alpha coefficient of 0.82.

The cardiotocographer (Contec CMS800G) performed NST. The pregnant women’s BP was measured using a manual arm BP monitor. A pulse oximeter was used to measure participants’ HR and SpO2.

### Data analysis

The quantitative variables are described using means and standard deviation (SD), or medians and interquartile range. The qualitative variables were described in absolute (n) and relative (%) frequencies. In order to test for differences in measurements over time and differences depending on the presence or absence of music, repeated-measures analysis of variance (ANOVA) was used. With the above method, the degree of change over time of the parameters under study was also assessed, depending on the presence or absence of music. Significance levels were two-sided, and statistical significance was set at p<0.05. The statistical program SPSS 22.0 was used for the analysis.

## RESULTS

### Characteristics of the participants

The participants’ mean age was 32.5 years (SD=4.2). The majority of the participants were married (94.7%), Greek (92.1%), had a higher education level (65.8%), and were primigravidas (73.7%). For all the participants, pregnancy was desired, and for 60.5% it was planned. Almost all of the participants intended to give birth by vaginal birth (97.4%). All participants attended antenatal classes and intended to breastfeed their infant (Table 1).

**Table 1:** Participant characteristics

*Characteristics*	*Categories*	*n*	*%*
**Age** (years), mean (SD)		32.5 (4.2)	
**Marital status**	Married	36	94.7
	Unmarried	2	5.3
**Nationality**	Greek	35	92.1
	Other	3	7.9
**Education level**	12 years	6	15.8
	Higher education	25	65.8
	Postgraduate or Doctoral degree	7	18.4
**Employment status**	Employed	37	97.4
	Unemployed	1	2.6
**Previous deliveries**	0	28	73.7
	1	8	21.1
	2	1	2.6
	3	1	2.6
**Number of miscarriages**	0	36	94.7
	1	2	5.3
**Planned pregnancy**	Yes	23	60.5
	No	15	39.5
**Intention of type of delivery**	Vaginal birth	37	97.4
	Cesarean section	1	2.6

### Anxiety levels of the participants

There were no significant pre-examination differences in the state anxiety levels whether they listened to music (mean=33.00, SD=6.84) or not (mean=33.50, SD=6.88, difference between groups p=0.615). Participants’ state anxiety with (mean=27.87, SD=4.55) or without music intervention (mean=31.16, SD=7.74) showed a significant reduction after the completion of the NST (difference between groups p=0.009). However, the reduction was significantly greater with the music intervention (difference between measurements p<0.001). Thus, in the post-test measurement, the state anxiety score of the women who had undergone the music intervention was significantly lower compared to those without the music intervention (Table 2).

**Table 2:** State anxiety scale score variation of pregnant women

	*State anxiety scale score*						*p^b^*	*p^c^*
	*Before NST*		*After NST*		*Variation*			
*Examination with music*	*Mean*	*SD*	*Mean*	*SD*	*Mean*	*SD*
No	33.00	6.84	31.16	7.74	-1.84	5.05	0.031	0.002
Yes	33.50	6.88	27.87	4.55	-5.63	5.18	<0.001	
p^a^	0.615		0.009					

a Difference between groups.

b Difference between measurements.

c Repeated measurements ANOVA. Differences in change from one measure to another between groups. NST: non-stress test.

Levels of trait anxiety before the NST (mean=40.97, SD=5.58) and after that (mean=40.50, SD=5.56) did not change significantly when there was no music intervention (difference between measurements p=0.344). These levels before the NST (mean=39.66, SD=5.44) and after its completion (mean=38.00, SD=5.39) showed a significant reduction when there was music intervention (difference between measurements p<0.001). The presence or absence of music substantially impacted the degree of variance in trait anxiety levels; when music was present, the degree of variance was decreased, and when there was no music, it remained stable (Table 3).

**Table 3:** Trait anxiety scale score variation of pregnant

	*Trait anxiety scale score*						*p^b^*	*p^c^*
	*Before NST*		*After NST*		*Variation*			
*Examination with music*	*Mean*	*SD*	*Mean*	*SD*	*Mean*	*SD*
No	40.97	5.58	40.50	5.56	-0.47	3.05	0.344	0.020
Yes	39.66	5.44	38.00	5.39	-1.66	2.59	<0.001	
p^a^	0.005		<0.001					

a Difference between groups.

b Difference between measurements.

c Repeated measurements ANOVA. Differences in change from one measure to another between groups. NST: non-stress test.

### Participants’ systolic and diastolic blood pressure

Participants’ SBP did not differ significantly between the examination with music (mean=101.2, SD=10.1) or without music intervention (mean=102, SD=12.2) at baseline (difference between groups p=0.657). The same results were observed at 8 minutes (mean=98.7, SD=9.3 vs mean=99.7, SD=11.1, difference between groups p=0.444). At 12 minutes, participants’ SBP was significantly lower when there was the music intervention (mean=93.2, SD=16.4 vs mean=99.5, SD=10.7, difference between groups p=0.030), as well as at 20 minutes (mean=93, SD=8 vs mean=100, SD=9.7, difference between groups p<0.001). During the NST performance, participants’ SBP remained stable when there was no music intervention (difference between measurements p=0.265), while when it was present, it decreased significantly at 12 and 20 minutes compared to the initial measurement (difference between measurements p<0.001). The degree of variation in SBP differed significantly between examinations with and without music (repeated measurements ANOVA p=0.001) (Table 4).

**Table 4:** Vital signs variations of pregnant women

*Vital signs*	*Examination with music*	*Initial measurement*	*8 min*	*12 min*	*20 min*	*p^b^*	*p^c^*
		*Mean (SD)*	*Mean (SD)*	*Mean (SD)*	*Mean (SD)*		
SBP	No	102 (12.2)	99.7 (11.1)	99.5 (10.7)	100 (9.7)	0.265	0.001
	Yes	101.2 (10.1)	98.7 (9.3)	93.2 (16.4)	93 (8)	<0.001	
	p^a^	0.657	0.444	0.030	<0.001		
DBP	No	57.8 (7.4)	56.6 (6.1)	56.7 (7.2)	56.7 (6.1)	0.711	0.026
	Yes	56.4 (5.4)	55.9 (6.2)	53.8 (6.1)	52.6 (6.8)	<0.001	
	p^a^	0.230	0.405	0.017	<0.001		
HR	No	84.9 (11.4)	85.1 (9.7)	85.8 (9.3)	84.6 (10.5)	0.762	0.328
	Yes	85.9 (10.4)	85.9 (9.9)	83.6 (8.8)	84.4 (9.7)	0.230	
	p^a^	0.506	0.611	0.113	0.909		
SpO2	No	95.7 (1.6)	95.5 (1.5)	95.9 (1.5)	95.6 (1.3)	0.461	0.380
	Yes	95.9 (1.5)	94.8 (4.1)	95.5 (1.7)	95 (3.8)	0.173	
	p^a^	0.453	0.282	0.234	0.370		

a Difference between groups.

b Difference between measurements.

c Repeated measurements ANOVA. Differences in change from one measure to another between groups. NST: non-stress test. SDB: systolic blood pressure. DBP: diastolic blood pressure. HR: heart rate. SpO2: peripheral oxygen saturation.

Participants’ DBP did not differ significantly between the examination with music (mean=56.4, SD=5.4) or without music intervention (mean=57.8, SD=7.4) at baseline (difference between groups p-value = 0.230). The same results were observed at 8 minutes (mean=55.9, SD=6.2 vs mean=56.6, SD=6.1, difference between groups p=0.405).

At 12 minutes, participants’ DBP was significantly lower when there was the music intervention (mean=53.8, SD=6.1 vs mean=56.7, SD=7.2, difference between groups p=0.017), as well as at 20 minutes (mean=52.6, SD=6.8 vs mean=56.7, SD=6.1, difference between groups p<0.001). During the NST performance, participants’ DBP remained stable when there was no music intervention (difference between measurements p=0.711), while when it was present, it decreased significantly at 12 and 20 minutes compared to the initial measurement (difference between measurements p<0.001). The degree of variation in DBP differed significantly between examinations with and without music intervention (repeated measurements ANOVA p=0.026) (Table 4).

### Participants’ heart rate and oxygen saturation

Participants’ HR did not differ significantly between the examination with music (mean=85.9, SD=10.4) or without music intervention (mean=84.9, SD=11.4) at baseline (difference between groups p=0.506). The same results were also observed at 8 minutes (mean=85.9, SD=9.9 vs mean=85.1, SD=9.7, difference between groups p=0.611), at 12 minutes (mean=83.6, SD=8.8 vs mean=85.8, SD=9.3, difference between groups p=0.113), and at 20 minutes (mean=84.4, SD=9.7 vs mean=84.6, SD=10.5, difference between groups p=0.909). Without the music intervention, participants’ HR was similar at all time intervals recorded (difference between measurements p=0.762). The music intervention also observed similar results regarding pregnant ’women’s HR (difference between measurements p=0.230). There was no significant variation in their values during the NST performance (Repeated measurements ANOVA p=0.328) (Table 4). Participants’ SpO2 did not differ significantly between the examination with music (mean=95.9, SD=1.5) or without music intervention (mean=95.7, SD=1.6) at baseline (difference between groups p=0.453). The same results were also observed at 8 minutes (mean=94.8, SD=4.1 vs mean=95.5, SD=1.5, difference between groups p=0.282), at 12 minutes (mean=95.5, SD=1.7 vs mean=95.9, SD=1.5, difference between groups p=0.234), and at 20 minutes (mean=95, SD=3.8 vs mean=95.6, SD=1.3, difference between groups p=0.370). Without the music intervention, participants’ SpO2 was similar at all time intervals recorded (difference between measurements p=0.461). The music intervention also observed similar results regarding pregnant women’s SpO2 (difference between measurements p=0.173). There was no significant variation in their values during the NST performance (repeated measurements ANOVA p=0.380) (Table 4).

### Fetal parameters

Fetal heart rate (FHR) was similar regardless of the presence or absence of music in the first 10 minutes (difference between groups p=0.871) and the last 10 minutes (difference between groups p=0.997). FHR was similar at all time intervals recorded with the music intervention (difference between measurements p=0.441) or without it (difference between measurements p=0.625). There was no significant difference in change from one measurement to another between the presence or absence of music (repeated measurements ANOVA p=0.826). Fetal movement (FM) increased significantly more in the last 10 minutes of the NST compared to the first 10 minutes, only when the NST was performed without music intervention (difference between measurements p=0.048). On the contrary, FM was similar at all time intervals recorded with the music intervention (difference between measurements p=0.973). There was no significant observation regarding the difference in change from one measurement to another between the presence or absence of music (repeated measurements ANOVA p=0.292). Baseline variability was similar regardless of the presence or absence of music in the first 10 minutes (difference between groups p=0.428) and the last 10 minutes (difference between groups p=0.409). Also, it was similar at all time intervals recorded with the music intervention (difference between measurements p=0.700) or without it (difference between measurements p=0.664), and there was no significant difference in change between measurements regarding the presence or absence of music (repeated measurements ANOVA p=0.963). Accelerations were similar regardless of the presence or absence of music in the first 10 minutes (difference between groups p=0.235) and the last 10 minutes (Difference between groups p=0.128). However, they increased significantly more in the last 10 minutes of the NST compared to the first 10 minutes, only when the NST was performed without music intervention (difference between measurements p=0.019). This finding was not observed with the music intervention since accelerations were similar at all time intervals recorded (difference between measurements p=0.246). The degree of variation in accelerations differed significantly depending on the presence or absence of music (repeated measurements ANOVA p=0.019). Decelerations were similar regardless of the presence or absence of music in the first 10 minutes (difference between groups p=0.324) and the last 10 minutes (difference between groups p=0.171). Also, they were similar at all time intervals recorded with the music intervention (difference between measurements p=0.324) or without it (difference between measurements p=0.171), and there was no significant difference in change between measurements regarding the presence or absence of music (repeated measurements ANOVA p=0.092). Uterine contractions were also similar regardless of the presence or absence of music in the first 10 minutes (difference between groups p=0.42) and the last 10 minutes (difference between groups p=0.137). Likewise, they were similar at all time intervals recorded with the music intervention (difference between measurements p=0.894) or without it (difference between measurements p=0.250), and there was no significant difference in change between measurements regarding the presence or absence of music (repeated measurements ANOVA p=0.228) (Table 5).

**Table 5:** Basic fetal parameters in both NST examinations of pregnant women

*Parameters*	*Examination with music*	*First 10 minutes of NST*		*Last 10 minutes of NST*		*p^b^*	*p^c^*
		*Mean (SD)*	*Median (IQR)*	*Mean (SD)*	*Median (IQR)*		
**Fetal movements**	No	6.4 (5.7)	5 (2–10)	8.1 (5.9)	7 (3–13)	0.048	0.292
	Yes	7.3 (6)	6.5 (3–10)	8.3 (10.5)	5 (2–13)	0.973	
	p^a^	0.598		0.383			
**Baseline fetal heart rate**	No	137.6 (9.1)	135 (130–145)	137.1 (8.2)	135 (130–145)	0.625	0.826
	Yes	138 (10.7)	137.5 (130–145)	137.2 (10.2)	140 (130–145)	0.441	
	p^a^	0.871		0.997			
**Baseline variability**	No	7.5 (2.5)	7.5 (5–10)	7.8 (2.8)	10 (5–10)	0.664	0.963
	Yes	7.1 (2.8)	5 (5–10)	7.2 (2.5)	5 (5–10)	0.700	
	p^a^	0.428		0.409			
**Accelerations**	No	1.8 (1.5)	2 (1–3)	2.6 (1.7)	3 (1–4)	0.019	0.019
	Yes	2.4 (1.9)	2 (1–3)	2.1 (2.2)	2 (0–3)	0.246	
	p^a^	0.235		0.128			
**Decelerations**	No	0 (0)	0 (0–0)	0.1 (0.4)	0 (0–0)	0.171	0.092
	Yes	0 (0.2)	0 (0–0)	0 (0)	0 (0–0)	0.324	
	p^a^	0.324		0.171			
**Uterine contractions**	No	0.3 (0.6)	0 (0–1)	0.5 (0.8)	0 (0–1)	0.250	0.228
	Yes	0.3 (0.5)	0 (0–1)	0.3 (0.6)	0 (0–0)	0.894	
	p^a^	0.42		0.137			

a Difference between groups.

b Difference between measurements.

c Repeated measurements ANOVA. Differences in change from one measure to another between groups. NST: non-stress test. IQR: interquartile range.

## DISCUSSION

The present study was conducted to determine the effect of NST on pregnant women’s anxiety levels and the effect of music during the NST performance on maternal anxiety levels, pregnant women’s vital signs, and fetal parameters. The study revealed that maternal anxiety levels decreased after the NST’s completion, regardless of the presence or absence of music. However, this reduction was more significant after the music intervention. Music only affected participants’ BP, and it was also observed that music contributed to maintaining FM and FHR accelerations at consistent levels.

The current study found that anxiety symptoms were reduced after the completion of the NST. This finding is consistent with those of Kafali et al.^27^, where a decrease in STAI score was observed in the study group. In the study by Erkun Dolker et al.^20^, a difference in the mean STAI score, which was lower for the control group after completing the NST, was also noted. The NST is probably a source of anxiety for pregnant women, and perhaps that is why we noticed differences in their anxiety levels after completing it. To the best of our knowledge, there is no extensive literature characterizing the NST as a potential source of anxiety. However, authors of previous studies^12,27^ have expressed that the NST process is anxiogenic. Additionally, some studies show that other prenatal non-invasive testing methods can affect the levels of anxiety of pregnant women^28,29^. The current findings cannot be safely generalized because they only provide a partial picture of the effect of the NST on anxiety levels due to the unknown pregnant women’s anxiety levels during either the previous NSTs or at the start of the third trimester.

Compared to the NST with no music intervention, the current study’s results suggest that STAI scores significantly decreased following the NST with music intervention in both the state and trait subscales. Our results are in agreement with previous studies^12,27^ but are in contrast to another which showed that music did not affect maternal anxiety levels^20^. It is likely that knowledge and experience of the NST procedure from a previous pregnancy play a role and are related to pregnant women’s anxiety levels after the NST completion. The current findings are not sufficiently indicative of the effect of music on maternal anxiety levels, and thus, it is not appropriate to generalize from them; yet, there are signs of this impact.

The current study found that not all of the pregnant women’s vital signs were affected by music. In particular, there was no discernible difference in the participants’ SpO2 and HR during the NST, regardless of whether the music intervention was present. This outcome contrasts with the study by García González et al.^21^, who reported a statistically significant decrease in HR among women receiving musical stimulation. Regarding participants’ BP, the current study found that it was significantly lower when there was a music intervention. Compared to the NST without music, the 12-minute recording’s SBP and DBP marked a significant reduction. This result agrees with the findings of a previous study, which also found a statistically significant decrease in both SBP and DBP in pregnant women receiving music stimulation^21^. This finding has also been observed in other studies with a sample of patients with severe cerebral damage^30^ and with coronary heart disease^7^. Therefore, according to the above, the suggestion that the effects produced by musical vibrations trigger responses of the autonomic nervous system, such as changes in BP, is strengthened . According to the present results, music can affect BP without affecting HR or SpO2.

According to the present results, not all of the essential fetal characteristics of the NST were affected by the music during the NST performance. More specifically, no difference in FM was noticed in the NST while music was present. In contrast to our results are those of Kafali et al.^27^, where it was observed that the number of FM in the music group was significantly higher than that of the control group. According to the present baseline variability and FHR results, the music’s presence or absence had no impact. Our results agree with those of Khoshkholgh et al.^31^ but contrast with other studies^21,27^ since they observed that the baseline FHR of the music group was noticeably more significant than that of the control group. The present study found no difference in accelerations in the NST while music was present. However, a higher number of accelerations in the music group than in the control group has been observed in other studies^20,27,31^. In the present study, the presence or absence of music had no impact on decelerations. Our results are similar to those of the study by Kafali et al.^27^ and Küçükkelepçe et al.^32^ but are in contrast to other findings^20^. Regarding uterine contractions, the current findings showed that the presence or absence of the music had no impact on them. Our results are similar to those by Brillo et al.^22^, who reported that no significant differences were found in uterine contractility between their study groups.

### Limitations

The study has certain limitations. It is challenging to generalize the results of the current study due to its small sample size and quasi-experimental design with one group. The differences in each study’s design, sample size, culture of the pregnant women, types of music, or even the duration of exposure to the musical stimulus could all contribute to the discrepancy in the results regarding pregnant women’s anxiety levels, their vital signs, and basic fetal parameters. Another weakness is that neither correlations nor adjusted regression analyses were performed; thus, the findings’ strength is limited.

### Future research

Further research regarding the impact of music during the NST on prenatal anxiety symptomatology would be worthwhile. Future studies might include in their design the comparison of the anxiety levels of pregnant women who are exposed to music only during the period of NST visits with those of pregnant women who are exposed to music for a more extended period of time, including the period of NST visits. Also, it would be interesting if future studies examined the relationships between other factors, such as the number of pregnancies and the anxiety levels of pregnant women who listen to music during NST visits. It is recommended that more in-depth research be done on the effects of music during the NST performance on pregnant women’s vital signs and fetal parameters. More robust conclusions may be drawn about this issue if the data mentioned above are associated with other factors, such as pregnant women’s anxiety levels or music types.

## CONCLUSIONS

The current findings indicate that maternal anxiety levels decreased following both the music intervention and the completion of the NST. Only the participants’ BP was affected by music, and it was shown that music contributed to maintaining FM and FHR accelerations at consistent levels. The preliminary findings of the present non-randomized, quasi-experimental study, with one pre- and post-test group, indicate that music may be a potentially available option in midwifery.

## Data Availability

The data supporting this research are available from the authors on reasonable request.
